# Alphacoronaviruses in New World Bats: Prevalence, Persistence, Phylogeny, and Potential for Interaction with Humans

**DOI:** 10.1371/journal.pone.0019156

**Published:** 2011-05-12

**Authors:** Christina Osborne, Paul M. Cryan, Thomas J. O'Shea, Lauren M. Oko, Christina Ndaluka, Charles H. Calisher, Andrew D. Berglund, Mead L. Klavetter, Richard A. Bowen, Kathryn V. Holmes, Samuel R. Dominguez

**Affiliations:** 1 Department of Pediatrics, University of Colorado School of Medicine, Aurora, Colorado, United States of America; 2 Department of Microbiology, University of Colorado School of Medicine, Aurora, Colorado, United States of America; 3 U.S. Geological Survey, Fort Collins Science Center, Fort Collins, Colorado, United States of America; 4 Pinon Canyon Maneuver Site, Model, Colorado, United States of America; 5 Department of Microbiology, Immunology and Pathology, Colorado State University, Fort Collins, Colorado, United States of America; 6 Department of Biomedical Sciences, Colorado State University, Fort Collins, Colorado, United States of America; U.S. Naval Medical Research Center Detachment, United States of America

## Abstract

Bats are reservoirs for many different coronaviruses (CoVs) as well as many other important zoonotic viruses. We sampled feces and/or anal swabs of 1,044 insectivorous bats of 2 families and 17 species from 21 different locations within Colorado from 2007 to 2009. We detected alphacoronavirus RNA in bats of 4 species: big brown bats (*Eptesicus fuscus*), 10% prevalence; long-legged bats (*Myotis volans*), 8% prevalence; little brown bats (*Myotis lucifugus*), 3% prevalence; and western long-eared bats (*Myotis evotis*), 2% prevalence. Overall, juvenile bats were twice as likely to be positive for CoV RNA as adult bats. At two of the rural sampling sites, CoV RNAs were detected in big brown and long-legged bats during the three sequential summers of this study. CoV RNA was detected in big brown bats in all five of the urban maternity roosts sampled throughout each of the periods tested. Individually tagged big brown bats that were positive for CoV RNA and later sampled again all became CoV RNA negative. Nucleotide sequences in the RdRp gene fell into 3 main clusters, all distinct from those of Old World bats. Similar nucleotide sequences were found in amplicons from gene 1b and the spike gene in both a big-brown and a long-legged bat, indicating that a CoV may be capable of infecting bats of different genera. These data suggest that ongoing evolution of CoVs in bats creates the possibility of a continued threat for emergence into hosts of other species. Alphacoronavirus RNA was detected at a high prevalence in big brown bats in roosts in close proximity to human habitations (10%) and known to have direct contact with people (19%), suggesting that significant potential opportunities exist for cross-species transmission of these viruses. Further CoV surveillance studies in bats throughout the Americas are warranted.

## Introduction

Bats play important roles in maintaining and transmitting zoonotic viruses [1], [2], [3]. More than 99 different viruses have been detected in and/or isolated from bats of diverse species [2] (and C. Calisher, personal communication). Rabies virus and other lyssaviruses infect bats of many species, and Old World fruit bats (family Pteropodidae) are reservoirs for both Hendra and Nipah viruses [4], [5], [6]. Two newly discovered human reoviruses, Melaka virus and Kampar virus, associated with influenza-like illnesses in humans, may be transmitted from small flying foxes (fruit bats; *Pteropus hypomelanus*) based on the close phylogenetic relationships of these viruses to Pulau virus, a bat reovirus [7], [8]. Egyptian fruit bats (*Rousetttus aegyptiacus*) are known reservoirs of Marburg and certain ebolaviruses [9], [10].

In humans, domestic animals, and birds, coronaviruses are common respiratory and enteric pathogens, and several CoVs cause systemic disease. Among the 5 known human coronaviruses, HCoV-229E and HCoV-NL63 are alphacoronaviruses (formerly called group 1 CoVs), HCoV-OC43 and HCoV-HKU1 are betacoronaviruses (formerly group 2a), and the severe acute respiratory syndrome-related coronavirus (SARS-CoV) and SARS-like CoVs are also betacoronaviruses (formerly group 2b). The SARS pandemic of 2002–03 was caused by SARS-CoV, a zoonotic coronavirus recently emerged from horseshoe bats (suborder Microchiroptera, family Rhinolophidae, genus *Rhinolophus*) from different locations in southeastern China [11], [12]. Extensive worldwide surveillance of bats showed that bats carry an enormous diversity of CoVs [13], [14], [15], [16], [17], [18], [19], [20], [21], [22]. Phylogenetic analysis of complete genome sequences of coronaviruses from bats, humans, birds, and other vertebrates suggests that bats may be the reservoir hosts from which all coronavirus lineages originated [23], [24].

The potential for emergence of zoonotic viruses into the human population depends on the prevalence of the virus in its host species, host range mutations within viral quasispecies, and the degree to which the reservoir host interacts with humans. In 2006, we reported the first detection of alphacoronavirus RNA in feces of North American bats sampled in the Rocky Mountain region of Colorado [17]. Here we describe a much larger and more comprehensive study of coronavirus prevalence, epizootiology, geographic distribution, and persistence, as well as preliminary phylogenetic analysis of CoV genome sequences in bats in Colorado.

## Materials and Methods

### Ethics Statement

Capture, marking, and sampling of bats followed guidelines of the American Society of Mammalogists [25] and animal protocols were approved by the Institutional Animal Care and Use Committee of the U.S. Geological Survey, Fort Collins Science Center (‘*Standard Operating Procedure 01-01 for the Capture, Handling, Marking, Tagging, Biopsy Sampling, and Collection of Bats*’) and Colorado State University (CSU IACUC number 03-096A). Bats were captured under authority of a scientific collecting license (permit numbers: 07TR738A3, 08TR2010, and 09TR2010) issued by the Colorado Division of Wildlife.

### Sample Collection

**Figure 1:**
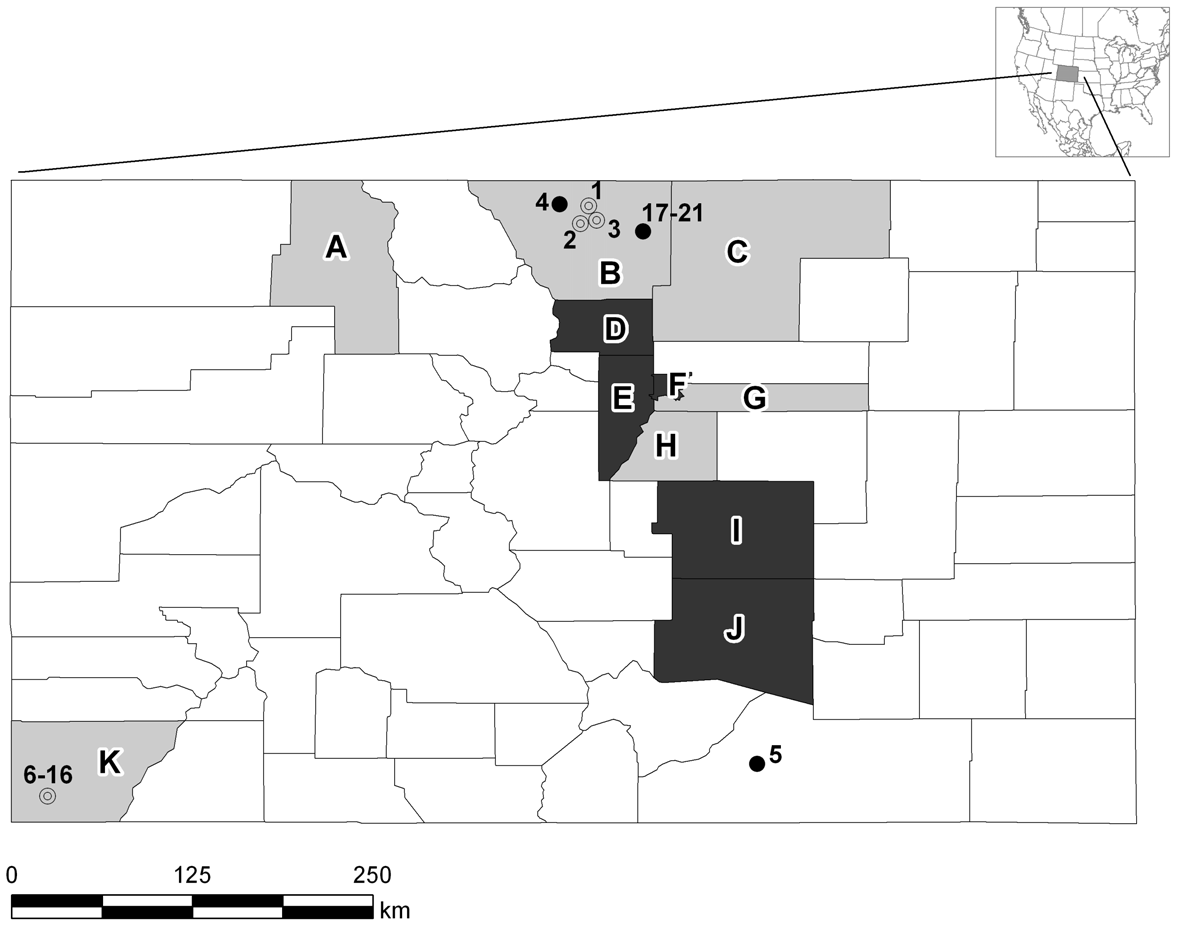
Map of Colorado showing sites where bats were sampled for the presence of CoV RNA. Circles (#1–21) represent sites where live bats were captured and fecal or swab samples were taken; closed circles represent sites where bats tested positive for CoV RNA and open circles are those from which all samples tested negative. Shaded counties (A–K) were those from which intestines of bats submitted to public health departments were sampled for CoV RNA. Counties from which intestinal samples were negative for CoV are shown in gray and counties with at least one CoV-positive intestinal sample are shown in black.

Insectivorous bats of the families Vespertilionidae (16 species) and Molossidae (1 species) were sampled at 16 rural sites (sites #1–16, Fig. 1) in the Rocky Mountain region during the summer of 2007. Bats were identified to species based on external morphological characteristics as described in regional faunal manuals [26], [27] adopting revised taxonomy for *Myotis occultus* [28] and *Parastrellus hesperus* [29]. To determine whether CoVs persist in bat populations over the course of several years, additional bat fecal samples were collected during the summers of 2008 and 2009 at two rural sites in north central and southeastern Colorado. In addition, big brown bats (*Eptesicus fuscus*) were sampled at 5 different sites (sites #17–21) within a single urban municipality in Northern Colorado (Fort Collins) during the summers of 2007 and 2008. These sites were chosen because they were in close proximity to humans [30]. Site #17 was in a vintage farmhouse that is currently being used as a family visitation center; site #18 was a natural creek surrounded by suburban neighborhoods; site #19 was in the recreation center of a church; site #20 was within an education building, and site #21 was within a picnic pavilion at a public park. Several of these sites had been previously used in rabies ecology studies, and some bats had been tagged with Passive Integrated Transponders (PIT tags) for host demographic analysis [31], [32]. This allowed for repeated capture and sampling of known individual bats.

All bats were either captured in mist nets during the night as they drank or foraged near open water, or were caught in mist nets or harp traps as they emerged from roosts. Whenever possible, the species, sex, reproductive status, age (adult or juvenile), date, and location of capture were recorded for each bat sampled. Bats were sampled as previously described [17], typically within 5–10 min of capture, and then released. Anal/rectal swabs or fecal pellets were taken using sterile calcium alginate swabs and stored in *RNA*later (Ambion, Austin, TX) and/or M4 viral transport medium (VTM, Remel; Lenexa, KS). All samples were stored at −70°C prior to analysis. Based on sample type and medium results were pooled for analysis of prevalence surveys. In a *post hoc* analysis we identified differences in the efficacy of different sampling methods (Text S1) such that the data represent minimal estimates of the prevalence of CoV infection in bats.

Bat carcasses submitted to the Colorado Department of Public Health and Environment (CDPHE) that were negative for rabies viruses were sent to our laboratory for detection of CoV RNA. These bats had been submitted from counties throughout Colorado for rabies testing to rule out the need for post-exposure rabies prophylaxis of humans who had had close contact with these animals [30], [33]. Intestines were removed from the bats and stored at −70°C prior to analysis.

### RNA Extraction and Reverse Transcription

For the 2007 samples, RNA from 200 µL of each sample was extracted on a Qiagen Biorobot EZ1 using the EZ1 viral RNA mini kit (Qiagen, Valencia, CA) according to the manufacturer's instructions. For all of the bat intestinal samples and all of the samples collected in 2008 and 2009, samples were homogenized with a Roche MagNALyser tissue homogenizer (Roche Applied Science, Indianapolis, IN) at a speed setting of 6000 for 20–40 seconds. RNA from 200 µL of each sample was extracted using the EZ1 RNA Universal Tissue Kit (QIAGEN, Valencia, CA). Extracted RNA was eluted in 60 µL of RNase-free water and stored at −80°C. Before RT-PCR, 50 microliters of RNA was treated with Zymo *OneStep* PCR Inhibitor Removal Kit (Zymo Research, Orange, CA) following the manufacturer's instructions. cDNA was generated by SuperScript III reverse transcriptase (Invitrogen, Carlsbad, CA) with random hexamers in a 20 µL reaction using 11 µL of RNA as a template according to the manufacturer's instructions. All samples were analyzed in duplicate. Reverse-transcription products were stored at −20°C.

### PCR and Nucleotide Sequencing

All cDNA samples collected from bats at rural sites or during 2007 were screened for CoV RNA by PCR with a pair of pan-CoV consensus primers [13] that amplify a highly conserved region (400 nucleotide amplicon) of the coronavirus RNA-dependent RNA polymerase (RdRp) gene as previously described [17] except that we used 2.0 µmol/L of primers and 1 µL of cDNA or PCR product (for hemi-nested reactions). To increase the sensitivity of RNA detection, based on our previously published bat CoV sequences [17] and new data from this study, we designed specific primers within the amplicons of alphacoronaviruses from bats of several species in the genus *Myotis* and big brown bats (Table S1). All of the specimens collected from long-legged and big brown bats were also tested with these primers.

To obtain longer nucleotide sequences, RT-PCR was performed using consensus degenerate primers from several regions within the RdRp gene in a SuperScript III one-step RT-PCR system with Platinum *Taq* High Fidelity kit (Invitrogen, San Diego, CA, USA). Similarly, we designed consensus primers that targeted a highly conserved region of the S2 region of the alphacoronavirus spike gene, and made primers from an exact S2 sequence obtained from a big brown bat (Table S1).

To minimize the possibility of contamination, all RT and PCR reactions were prepared in an enclosed acrylic nucleic acid workstation equipped with a UV light (Clone Zone, USA Scientific, Ocala, FL) in a room separate from the main laboratory. Water controls without template included in every RT and PCR experiment gave no false-positive results. Amplicons were analyzed by agarose gel electrophoresis and sequenced on an ABI 3730 DNA sequencer (Applied Biosystems Technologies, Carlsbad, CA) at the University of Colorado School of Medicine Cancer Center DNA Sequencing and Analysis Core. Samples were scored as positive if CoV RNA was detected on two PCR runs. Statistical significance was determined using Fisher's exact test. Phylogenetic analyses were conducted using MEGA version 4, and phylogenetic trees were constructed using the neighbor-joining method [34]. The nucleotide sequences from this study were deposited in GenBank under accession numbers HQ336973–HQ336976 and JF414933–JF414936.

## Results

### Prevalence of CoV RNA in Rocky Mountain Bats

A total of 983 fecal samples and/or anal region swabs and 61 intestinal samples was obtained from bats of 2 families and 17 different species during the summers of 2007–09. None of the trapped live bats sampled showed obvious signs of illness. CoV RNA was detected in 75 (7.2%) of the 1,044 samples from bats of 4 of the 17 species sampled (Table 1). The prevalence of CoV RNA was 12.0% (61 positive bats of 494 tested) for big brown bats (*Eptesicus fuscus*), 8.2% (12 positives of 147 sampled) for long-legged bats (*Myotis volans*), 3.2% (1 positive of 31 sampled) for little brown bats (*Myotis lucifugus*), and 1.9% (1 positive of 52 sampled) for western long-eared bats (*Myotis evotis*). CoV RNA was detected in bat samples from only 2 of the 16 rural locations, but in all five of the urban locations sampled (Figure 1).

**Table 1:** Prevalence of bats of various species that were positive* for CoV RNA at different sites.

Site #	Species	Total Tested	# Positive	% Positive
1	*Myotis lucifugus*	11	0	0
2	*Myotis volans*	3	0	0
3	*Eptesicus fuscus*	29	0	0
	*Lasionycteris noctivagans*	1	0	0
4	***Eptesicus fuscus***	**5**	**1**	**20**
	*Lasiurus cinereus*	20	0	0
	*Lasionycteris noctivagans*	1	0	0
	*Myotis ciliolabrum*	1	0	0
	***Myotis evotis***	**7**	**1**	**14**
	***Myotis volans***	**76**	**12**	**16**
5	*Antrozous pallidus*	11	0	0
	*Corynorhinus townsendii*	1	0	0
	***Eptesicus fuscus***	**18**	**8**	**44**
	*Lasiurus cinereus*	1	0	0
	*Lasionycteris noctivagans*	3	0	0
	*Myotis thysanodes*	3	0	0
	*Myotis yumanensis*	13	0	0
	*Parastrellus hesperus*	6	0	0
6	*Myotis occultus*	9	0	0
7	*Myotis yumanensis*	1	0	0
8	*Eptesicus fuscus*	2	0	0
	*Lasiurus cinereus*	1	0	0
	*Myotis californicus*	1	0	0
	*Myotis ciliolabrum*	1	0	0
	*Myotis evotis*	1	0	0
	*Myotis thysanodes*	1	0	0
	*Myotis volans*	4	0	0
9	*Eptesicus fuscus*	18	0	0
	*Euderma maculatum*	1	0	0
	*Lasiurus cinereus*	7	0	0
	*Lasionycteris noctivagans*	7	0	0
	*Myotis californicus*	7	0	0
	*Myotis ciliolabrum*	19	0	0
	*Myotis evotis*	22	0	0
	*Myotis thysanodes*	4	0	0
	*Myotis volans*	22	0	0
	*Myotis yumanensis*	3	0	0
	*Parastrellus hesperus*	8	0	0
	*Tadarida brasiliensis*	13	0	0
10	*Eptesicus fuscus*	8	0	0
	*Lasionycteris noctivagans*	13	0	0
	*Myotis evotis*	6	0	0
	*Myotis occultus*	7	0	0
	*Myotis volans*	23	0	0
	*Myotis yumanensis*	1	0	0
11	*Eptesicus fuscus*	3	0	0
	*Lasiurus cinereus*	7	0	0
	*Lasionycteris noctivagans*	3	0	0
	*Myotis volans*	11	0	0
12	*Antrozous pallidus*	2	0	0
	*Corynorhinus townsendii*	3	0	0
13	*Eptesicus fuscus*	2	0	0
	*Euderma maculatum*	2	0	0
	*Lasionycteris noctivagans*	3	0	0
	*Myotis evotis*	6	0	0
	*Myotis occultus*	6	0	0
	*Myotis volans*	4	0	0
14	*Eptesicus fuscus*	5	0	0
	*Lasiurus cinereus*	3	0	0
	*Lasionycteris noctivagans*	11	0	0
	*Myotis evotis*	3	0	0
	*Myotis thysanodes*	13	0	0
15	*Myotis evotis*	7	0	0
	*Myotis thysanodes*	1	0	0
16	*Myotis volans*	3	0	0
17	***Eptesicus fuscus***	**123**	**16**	**13**
18	***Eptesicus fuscus***	**29**	**5**	**17**
19	***Eptesicus fuscus***	**123**	**11**	**9**
20	***Eptesicus fuscus***	**41**	**4**	**10**
21	***Eptesicus fuscus***	**149**	**10**	**7**
CDPHE+	***Eptesicus fuscus***	**29**	**6**	**19**
	*Myotis ciliolabrum*	8	0	0
	*Myotis evotis*	3	0	0
	***Myotis lucifugus***	**20**	**1**	**5**
	*Myotis volans*	1	0	0
	**TOTAL**	**1044**	**75**	7
**ALL**	***Eptesicus fuscus***	**494**	**61**	**12**
	***Myotis evotis***	**52**	**1**	**2**
	***Myotis lucifugus***	**31**	**1**	**3**
	***Myotis volans***	**147**	**12**	**8**

*All bats at rural sites #1–16, all bats sampled during 2007 at urban sites #17–21, and all bats sampled from the CDPHE were tested for CoV RNA using the conserved coronavirus primer set. All bats from the genera *Myotis* and *Eptesiscus* were also screened with alphacoronavirus primer sets specific for these genera. Bats at urban sites #17–21 collected during 2008–09 were screened only with the species specific primer sets.

+ CDPHE = samples obtained from the Colorado Department of Public Health and Environment submitted from Arapahoe (1), Boulder (17), Denver (1), Douglas (1), El Paso (6), Jefferson (13), Larimer (1), Montezuma(1), Pueblo (10) , Routt (1), Weld (1), and unknown (8) counties. Bold type indicates CoV positive bat species. Subsequent tables show subsets of data from animals presented in this table.

The CDPHE provided 61 bats of 4 different species for testing of intestinal samples for CoV RNA. Of those sampled, 7 (11%) bats from 5 of the 11 Colorado counties sampled were positive for CoV RNA (Figure 1). Six (21%) of 29 big brown bats and 1 (5%) of 20 little brown bats tested were positive for CoV RNA (Table 1).

### Persistence of CoV RNA in Bat Populations

At site #4, a high-elevation meadow in a mountainous area of north-central Colorado, 76 long-legged bats were sampled during three consecutive summers (2007–2009). Although the sampled bats were not individually marked, the consistent capture of large numbers of females soon after sunset at the site indicated that most of the sampled bats likely came from a nearby maternity roost. Female bats often show year-to-year fidelity to maternity roosts [35]. The percentage of long-legged bats that tested positive for CoV RNA at site #4 varied by year from 6% to 31% (Table 2).

**Table 2:** Prevalence of coronavirus RNA in long-legged bats (site #4) or big brown bats (sites #5, 17–21) in fecal and/or anal swab samples by site of collection and date.

	Collection Date	Number of Bats Sampled	Number of Bats Positive for CoV RNA	% Positive	p value*
SITE #4					
	2007	16	5	31	
	2008	34	2	6	
	2009	26	5	19	
	**TOTAL**	**76**	**12**	**16**	**0.02** a
SITE #5	2008	4	4	100	
	2009	14	4	29	
	**TOTAL**	**18**	**8**	**44**	**0.02**
SITE #17					
	06-14-2007	31	0	0	
	08-14-2007	12	1	8	
	**2007 Total**	**43**	**1**	**2**	
	06-17-2008	23	2	9	
	07-08-2008	13	4	31	
	07-31-2008	44	9	20	
	**2008 Total**	**80**	**15**	**19**	
	**Site Total**	**123**	**16**	**13**	**0.01**
SITE #18					
	06-25-2008	26	3	12	
	07-07-2008	3	2	67	
	**2008 Total**	**29**	**5**	**17**	
	**Site Total**	**29**	**5**	**17**	
SITE #19					
	06-15-2007	40	3	8	
	08-22-2007	27	4	15	
	**2007 Total**	**67**	**7**	**10**	
	06-06-2008	25	2	8	
	07-01-2008	31	2	6	
	**2008 Total**	**56**	**4**	**7**	
	**Site Total**	**123**	**11**	**9**	**0.75**
SITE #20					
	06-20-2007	12	1	8	
	**2007 Total**	**12**	**1**	**8**	
	06-04-2008	13	2	17	
	06-23-2008	16	1	6	
	**2008 Total**	**29**	**3**	**10**	
	**Site Total**	**41**	**4**	**10**	**1.0**
SITE #21					
	06-19-2007	29	0	0	
	08-17-2007	22	0	0	
	**2007 Total**	**51**	**0**	**0**	
	06-03-2008	39	2	5	
	06-26-2008	24	4	17	
	08-05-2008	35	4	11	
	**2008 Total**	**98**	**10**	**10**	
	**Site Total**	**149**	**10**	**7**	**0.02**
SITES					
#17–21	**2007 Total**	**173**	**9**	**5**	
	**2008 Total**	**292**	**37**	**13**	
	**Site Total**	**465**	**49**	**10**	**<0.01**

*Fisher's exact test, comparisons between percent positive at indicated site between the two years sampled.

a comparison between 2007 and 2008, other comparisons not significant.

At site #5, an arid grassland bisected by canyons in southeastern Colorado [36], 56 bats of eight different species were sampled during two consecutive summers (2008 and 2009). Only big brown bats at site #5 were positive for CoV RNA. Although the number of big brown bats sampled at site #5 was small (4 in 2008 and 14 in 2009), the prevalence of CoV RNA in these bats during these two summers was high (29% to 100%) (Table 2).

In the five different urban locations (sites #17–21), 465 samples were collected from big brown bats during the summers of 2007 and 2008 (Table 2). Forty-six (10%) of the bats from these urban sites were positive for CoV RNA. The prevalence of CoV infection varied from 2%–19% depending on the site, month, and year of collection. CoV RNA was detected in bats from all of 5 roosts sampled during both summers. The prevalence of CoV RNA in bats was higher in 2008 (13%) than in 2007 (5%). During 2008, the prevalence of CoV RNA in big brown bats at individual sites tended to be higher during June and/or early July than later in the summer.

### Lack of Persistence of CoV Infections in Individually Tagged Big Brown Bats in Urban Roosts

All of the urban bat sampling sites were part of a previous study of the ecology of rabies in big brown bats that emphasized host demography [31], [32], and 113 (24%) of the 465 bats from these sites sampled for this study had been previously individually tagged. Sixteen (14%) of these tagged bats were captured and sampled more than once (14 captured twice, and 2 captured three times). Five (31%) of the 16 repeatedly sampled tagged bats captured in 2008 were positive for CoV RNA, but no CoV RNA could be detected in subsequent samples (Table 3). Four of the 5 bats became negative for CoV RNA within 6 weeks after they tested positive for CoV RNA. (The fifth bat was not recaptured after turning positive). Thus, in this small group of serially sampled bats, individual bats were not continually shedding detectable amounts of CoV RNA, so did not appear to be persistently infected.

**Table 3:** Detection of CoV RNA in 16 individually tagged big brown bats that were captured and sampled on multiple dates during the summer of 2008.

Sampling Site #	Bat	Sample 1	Sample 2	Sample 3
		**6/17/2008**	**7/8/2008**	**7/31/2008**
17	**1**	**+**	**NS**	**−**
	**2**	**NS**	**+**	**−**
	**3**	**−**	**NS**	**+**
		**6/3/2008**	**6/26/2008**	**8/5/2008**
21	**4**	**−**	**+**	**−**
	**5**	**+**	**−**	**NS**

All five of the positive bats were adult female big brown bats.

NS = not sampled, + = positive or CoV RNA, − = negative for CoV RNA.

### Age and Sex Distribution of Bats Positive for CoV RNA

The age and sex distributions of the 999 (94%) bats sampled for which these data were available and the subset of big brown bats in the urban maternity roosts sampled are shown in Table 4. Juvenile bats were two times more likely to be positive for CoV RNA than adults bats (13% vs. 6%, p = 0.008). In the urban maternity roosts, as expected, the majority of the big brown bats sampled were adult females, but juvenile bats (10 of 52 tested, 19%) were also more than twice as likely to be positive for CoV RNA than adult bats (36 of 413 tested, 9%, p = 0.03).

**Table 4:** Percent of bats positive for coronavirus RNA in fecal and/or anal swab samples by age and sex (N = 999).

Sex and Age	Number of Bats Tested	Number of Bats Positive for CoV RNA	% Bats Positive for CoV RNA	p value*
TOTAL				
Males	302	19	6	0.59
Females	697	52	8	
				
Adults	877	55	6	0.008
Juveniles	122	16	13	
				
Urban Maternity Roosts (all *Eptesicus fuscus* bats)				
Males	61	11	18	0.04
Females	404	35	9	
				
Adults	413	36	9	0.03
Juveniles	52	10	19	

*Fisher's exact test.

### Preliminary Phylogenetic Analysis of Rocky Mountain Bat CoVs

From the samples positive for CoV RNA, we obtained nucleotide sequences of amplicons ranging in length from 93–356 nt from the RdRp region of gene 1b. These formed three clusters (>90% nt identity within each cluster). The first cluster (A) included CoV RNAs of big brown bats from sites #5 and #17–21, the one big brown bat from site #4, and two long-legged bats from site #4 that were collected in 2007 and 2009. The sequence of the A cluster (representative bat: RM-Bt-CoV 453/2007 EF) was 96% identical to the same region from a big brown bat (RM-Bt-CoV 65) reported in our previous study [17]. The second cluster (B) (representative bat: RM-Bt-CoV 09-07/2009 MV) was found in 2 long-legged bats (one sampled in 2008 and one in 2009) and one western long-eared bat sampled at site #4. These sequences had >97% identity in this region to CoV RNA obtained from several occult bats (*M. occultus*; RM-Bt-CoV 6 and 11) reported previously [17]. The third cluster (C) of CoV amplicons (representative bat: RM-Bt-CoV 429/2007 MV) were from other long-legged bats sampled at site #4. These sequences were 96% identical to that from an occult bat (RM-Bt-CoV 3) reported previously (Table 5 and Figure S1). Cluster A had <65% identity with clusters B and C, whereas clusters B and C had 83% identity to one another.

**Table 5:** Nucleotide Sequence Comparison of New World Bat Coronavirus RNA in the RdRp region.

	RM-Bat CoV 453/2007/EF	RM-Bat CoV 433/2007/MV	RM-Bat CoV 09-07/2009MV	RM-Bat CoV 429/2007/MV	RM-Bat CoV CDPHE 15/ML	RM-Bat CoV CDPHE 61/EF	Trinidad bat CoV 1FY2/BA/2007	Trinidad bat CoV 1CO7/BA/2007
RM-Bat CoV 453/2007/EF(cluster A)	100 (309)	99 (309)	63 (182)	47. (309)	62 (182)	91 (210)	71 (309)	70 (309)
RM-Bat CoV 433/2007/MV(cluster A)		100 (350)	61 (182)	52 (332)	67 (182)	96 (210)	71 (309)	70 (350)
RM-Bat CoV 09-07/2009/MV(cluster B)			100 (182)	83 (182)	97 (182)	56 (182)	80 (182)	72 (182)
RM-Bat CoV 429/2007/MV(cluster C)				100 (182)	84 (182)	73 (210)	75 (332)	70 (332)
RM-Bat CoV 15/2006/ML(cluster B)					100 (3859)	72 (3410)	73 (1358)	72 (182)
RM-Bat CoV 61/2007/EF(cluster C)						100 (4012)	75 (1413)	76 (210)
Trinidad bat CoV 1FY2/BA/2007							100 (5160)	76 (3899)
Trinidad bat CoV 1CO7/BA/2007								100 (3905)

Results are shown as percent nucleotide identity. The sizes of the amplicons studies are shown in parenthesis.

**Figure 2:**
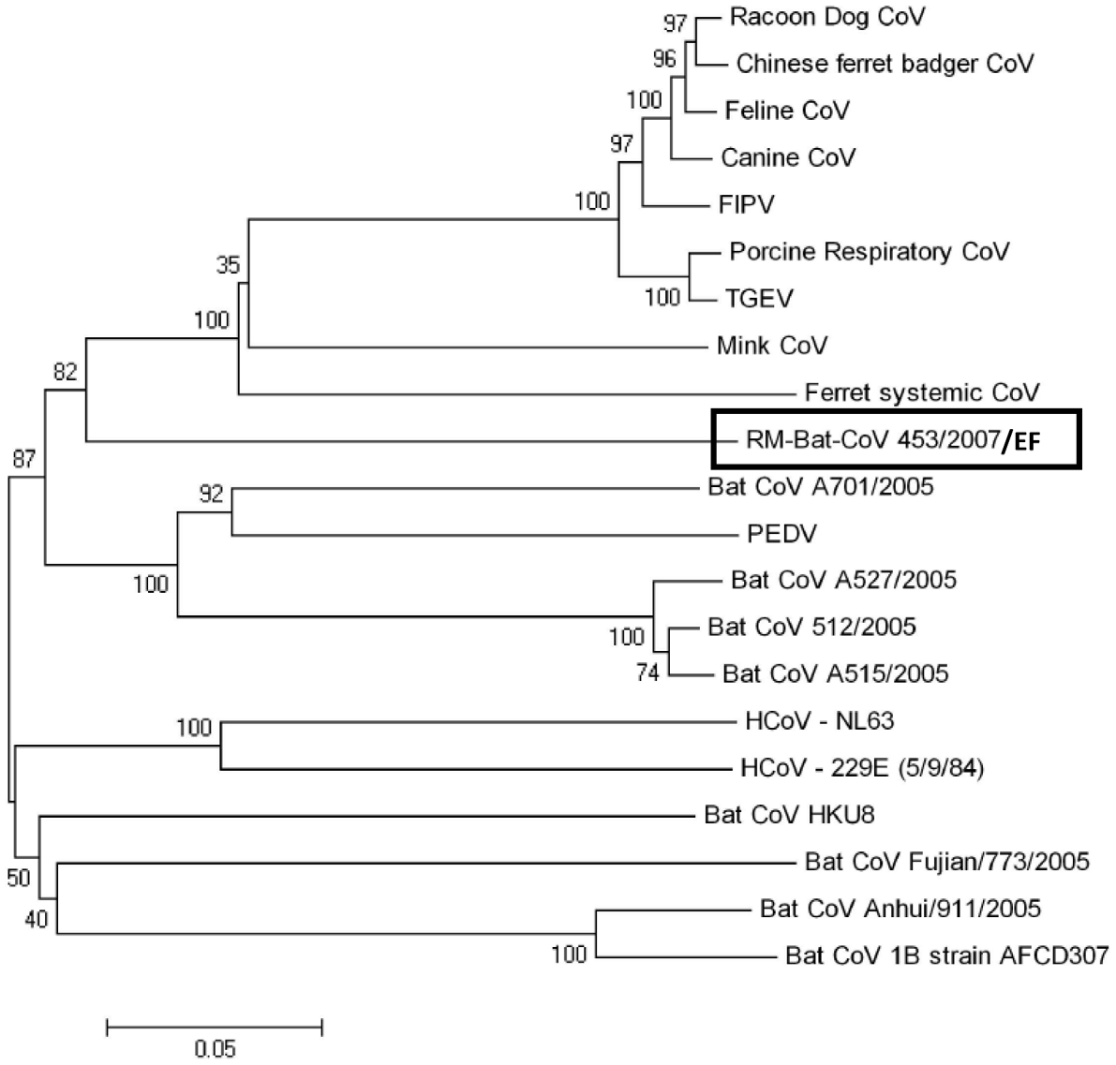
Phylogenetic Analysis of the spike gene. Phylogenetic analysis of an 1100 nucleotide segment of the S2 region of the spike gene of RM-Bat-CoV 453/2007/EF (*Eptesicus fuscus*) compared to other known alphacoronaviruses. Phylogenetic trees were constructed by the neighbor-joining method using MEGA version 4.

An 1100 nt sequence encoding the S2 domain of the spike glycoprotein was obtained from a big brown bat collected at site #4 in 2007 (Rocky Mountain Bat-CoV 453/2007 EF). We compared this sequence to S2 sequences of other known coronaviruses (Table 5 and Figure 2) and found that this genome was distantly related to other known alphacoronaviruses in group 1a, with <67% nucleotide identity to CoVs. We also obtained a 700 nucleotide sequence in the same region of S2 from the long-legged bat (RM-Bat-CoV 433/2007 MV) that had a similar sequence to this big brown bat in the RdRp gene (both in RdRp cluster A). These S2 amplicons had >98% nt sequence identity. The closest bat coronavirus spike sequence to RM-Bt-CoV 453/2007 found in GenBank, was Bt-CoV A701, from an Old World species, Rickett's big-footed bat (*Myotis ricketti*) sampled in Southeast China in 2005 [14] (65% nucleotide identity, 65% amino acid identity).

**Figure 3:**
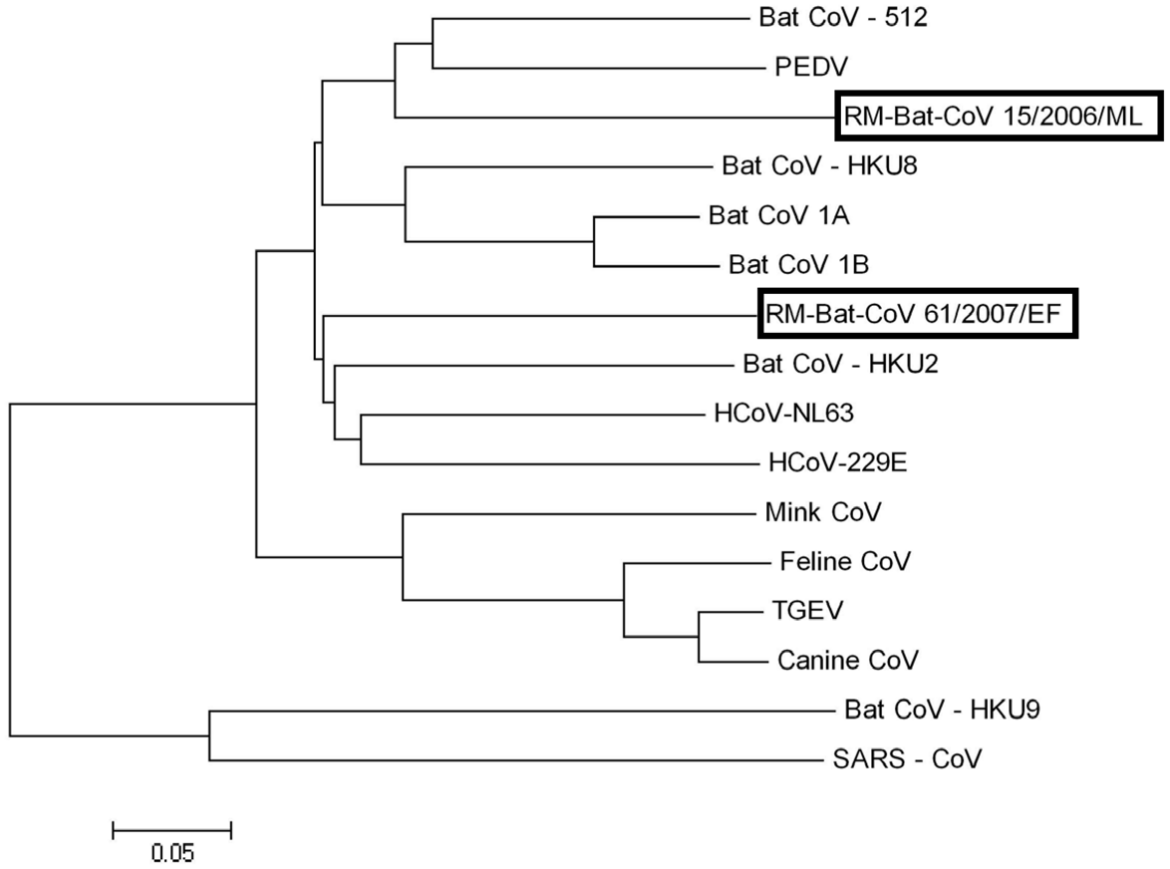
Phylogenetic analysis of the RdRp gene. Phylogenetic analysis of an approximate 4000 nucleotide sequence (2 segments) of the RdRp gene of RM-Bat-CoV-15/2006/ML (*Myotis lucifugus*) and RM-Bat-CoV 61/2007/EF (*Eptesicus fuscus*) compared to other known coronaviruses. Phylogenetic trees were constructed by the neighbor-joining method using MEGA version 4.

An approximately 4000 nt sequence in 2 segments of the RdRp gene was obtained from one of the little brown (RM-Bt-CoV-15/2006/ML) and one of the big brown bats (RM-Bt-CoV-61/2007/ EF) that were submitted to the CDPHE. These nt sequences were only 62% identical, indicating that they represented two unique viruses in bats of these two species. These sequences were distantly related (<75% nt identity) to other known alphacoronaviruses, with <75% nt identity to CoVs in this group, including all currently available Old World bat CoVs (Table 5 and Figure 3).

## Discussion

This is the first multiyear surveillance project of CoVs in wild bats in North America. CoV RNA was detected in approximately 7% of all bats sampled (likely an underestimate of prevalence, Text S1), comparable to the prevalence of CoV RNA detected in various species of bats reported in other parts of the world (ranging from 2–55%) [14], [18], [19], [21], [22], [37], [38], [39]. In our study no CoV RNA was detected in bats in 13 of the 17 species we sampled (also likely biased negatively). Failure to detect CoVs in bats of these species could be related to the smaller numbers sampled. However, a relatively high prevalence of CoV RNA was detected in bats of 2 species collected at several different sites: 12% for big brown bats and 8% for long-legged bats, and at lower prevalence, 3% in little brown bats and 2% in western long-eared bats.

In marked contrast to the enormous diversity of CoV genomes found in Old World bats [14], [24], [40], in this and several other CoV surveillance studies of New World bats [17], [18], [22], all CoVs detected were alphacoronaviruses. Our data indicate that nucleotide sequences of alphacoronaviruses harbored by Colorado bats are distinct from those found in Old World bats. Two recent studies of the bat guano virome using next generation sequence technology also only detected alphacoronaviruses in the New World bats of the species tested, as well as a diverse array of other types of viruses [41], [42]. Thus, so far there appears to be much more limited CoV diversity in New World bats of the species tested than in Old World bats.

Betacoronaviruses have only been detected in Old World bat species belonging to the families Pteropodidae (*Rousettus* spp) and *Rhinolophidae* (*Rhinolophus* spp.) which belong to the chiropteran suborder Yinpterochiroptera. Based on available evidence, betacoronaviruses could be restricted to hosts in the suborder Yinpterochiroptera (families Pteropodidae, Rhinolophidae, Megadermatidae, Craseonycteridae, Rhinopomatidae). No bat families of the suborder Yinpterochiroptera occur in the New World. [43]. The finding of only alphacoronaviruses in our study may be because bats of these species are resistant to other CoVs and/or bats from different parts of the New World have yet to be tested for CoV infection, as we sampled bats from only a subset of the hundreds of species that reside in the New World.

These observations also support the hypothesis that coronaviruses may have co-evolved with their bat hosts, as no species of bat is found both in the New World and Old World [44]. To date, however, only a small subset of New World species of bats has been tested for coronavirus infection. As 75% of living genera of all bats worldwide are found in the New Worlds tropics alone, further CoV surveillance in bats of additional species from different regions in the Western hemisphere may reveal hitherto undetected varieties of coronaviruses.

The seasonal epidemiology and persistence of New World CoV infections in individual bats and within bat populations has not been elucidated. The most comprehensive epidemiological investigation of CoVs to date in Old World bat populations showed that the prevalence of SARS-Rh-BatCoVs in rhinolophid bats over a four-year period at collection sites in Hong Kong SAR and China peaked in the spring and varied from year to year. We found similar results in New World bats. At site #4 long-legged bats had an alphacoronavirus RNA prevalence of 31% in 2007, 19% in 2009, but only 6% in 2008. In all five of the urban maternity roosts sampled, CoVs persisted in bat roosts throughout the course of the non-hibernating part of the year (spring/summer) and persisted from year to year. We also found that the prevalence of CoV infection in these bat roosts tended to peak in late spring/early summer. The prevalence of infection with human CoVs also shows significant annual variations [45], possibly depending on environmental conditions and/or fluctuating CoV antibody levels in the population. Possible seasonal variation in CoV infection rates may explain why in our initial 2006 study we found a high prevalence (50%) of alphacoronavirus RNA in occult bats [17], but in 2007 we did not detect any positive individuals (22 tested in the same region).

The majority of the bats sampled in our study were adult females because they were primarily captured from maternity roosts. The highest prevalence of infection was noted in juvenile bats. In Germany, CoV infection was also found to be associated with young age and was more common in female bats from maternity roosts compared to female bats found at foraging or swarming sites [19]. These findings support the hypothesis that younger bats may be more susceptible to CoV infection and may serve to propagate and maintain these viruses within bat colonies.

No overt clinical manifestations of disease were observed in any of the captured bats, including those that were infected with CoVs. In the small subset of bats that were tagged and recaptured, no individual bat remained persistently positive for CoV RNA after 6 weeks. Similar findings were made in rhinolophid bats in Asia that harbor SARs-like-bat-CoVs [37] and in fruit bats experimentally infected with bat CoVs which showed no signs of illness [39]. These data suggest that although CoVs persist within bat populations, individual bats may experience only self-limited infections with CoVs without apparent illness.

Phylogenetic studies of CoV genomes in Old World bats in Asia and Europe have suggested that some bat CoVs may infect bats of only one species or several closely related species. In Asia and Germany, different species of bats roosting in the same cave were found to host different CoVs, whereas bats of the same species in different locations harbored similar CoVs [14], [19]. In Europe, strict associations were found between bat CoV deduced amino acid sequences in an 816 bp fragment of the RdRp gene and their specific bat hosts [40]. In Africa, CoVs found in one species of bat were not detected in bats of different species co-roosting in the same cave [38]. Similarly, our study showed that New World bats of the same species in geographically distinct locations and over the course of several years harbor similar CoVs. In contrast to these findings, in Kenya some CoVs appear to be able to infect Old World bats of several different species [21]. Our preliminary nucleotide sequence data also suggests that we found very closely related CoV nucleotide sequences in New World bats from three different species of *Myotis* (*M. volans*, *M. evotis*, and *M. occultus*). Furthermore, in site #4, we found similar nucleotide sequences in the spike and replicase genes in CoV RNAs from both a big-brown bat and a long-legged bat, suggesting that at least some New World bat CoVs may be able to infect bats of different genera. These findings are notable, as recent phylogenetic studies of rabies viruses in bats suggest that host species barriers play a key role in cross species transmission of viruses [46].

To assess the potential for zoonotic transmission of bat CoVs, we focused part of this present work on North American bats that have the closest contact with humans and sampled roosts where big brown bats had histories of contact or potential for contact with people [30]. Big brown bats are common inhabitants of buildings in cities and towns in Colorado and across the United States, and are the primary species encountered by humans in terms of potential exposure to disease agents [30], [33], [47] These bats had a high prevalence of CoV infection, ranging from 0–67% (overall 10%) depending on the site and time of year. Big brown bats submitted to the CDPHE for rabies testing because of known direct contact with humans also had a very high prevalence (19%) of CoV infection. Because bats which have known or potential contact with humans have such a high prevalence of CoV infection, opportunities exist for potential transmission of these viruses to humans.

Following the SARS epidemic, intensive surveillance detected a great diversity of CoVs throughout the animal kingdom. CoVs can undergo a high frequency of RNA recombination, both *in vitro* and *in vivo*, which may play an important role in their evolution and virulence [48]. Old World bat CoVs of several different genotypes were found to co-exist in a single bat [49]. Thus recombination between different bat CoVs could potentially occur *in vivo*, giving rise to new CoV genomes. Two strains of HCoV- HKU1 have recombined to yield a novel HCoV-HKU1 genotype [16], and recombination between different strains of SARS-CoV-like viruses in bats may have given rise to civet SARS-CoV [37]. The great diversity of CoVs, their high frequency of RNA recombination, their ability to persist in bat populations, and the finding that some CoVs can apparently infect bats of divergent genera, suggest that ongoing evolution of CoVs in bats may pose a continuing threat for emergence of novel CoVs into new hosts.

## Supporting Information

Figure S1 **Sequence alignment of representative samples of the 1b gene obtained in this study (2007–09 collection) compared with sequences obtained from bats collected in previous study (2006 collection).** **A.** Amplicons obtained from a big brown bat (07-453 EF) and a long legged (07-433 MV) bat in 2007 have 97% sequence similarity with a big brown bat (RM Bt-CoV 65) collected in 2006. **B.** Amplicon from a long legged bat (07-607 MV) is most similar to amplicons obtained from an occult myotis bat (RM-Bt-CoV 48), but with only 85% similarity.
(DOC)

Table S1 **Primers and RT-PCR programs.** **A.** Consensus primers targeted a highly conserved region of the S2 region of the spike gene and from an exact sequence obtained from one of the big brown bats. PCR was performed under the following conditions: one µL of cDNA was amplified in a 50-µL reaction containing, 0.2 µmol/L deoxynucleoside triphosphates, 1 U of PhusionTaq High-Fidelity DNA Polymerase (Finnzymes, Espoo, Finland), and 2.0 µmol/L primers by the following PCR program: 30 sec at 98°C; 40 cycles for 10 sec at 98°C, 15 sec at 50–52°C (depending on the primer set), and 15 sec at 72°C; and then 10 min at 72°C. **B.** Primers used for detection of CoV sequence in bat samples. One microliter of cDNA was amplified in a 50-µL reaction containing 1.5 mmol/L MgCl_2_, 0.2 µmol/L deoxynucleoside triphosphates, 2.5 U of HotStarTaq (QIAGEN), and 2.0 µmol/L primers using the following PCR program: 15 min at 95°C; 45 cycles for 1 min at 95°C, 1 min at 48°C for MY-F and MY-R and 50°C for EF-F and EF-R, and 1 min at 72°C; and 10 min at 72°C. **C.** To obtain additional sequences for phylogenetic analysis, for two of the CDPHE intestinal samples, RT-PCR was performed using consensus degenerate primers from several areas within the RdRp gene in a SuperScript III one-step RT-PCR system with Platinum *Taq* High Fidelity kit (Invitrogen, San Diego, CA, USA). Primers and protocols were kindly provided by Suxiang Tong, PhD and Ying Tao, PhD of the Centers for Disease Control and Prevention, Atlanta, Georgia, USA.
(DOC)

Text S1 **Influence of different sampling and analysis techniques on CoV RNA detection.**
(DOC)
