# Supplementary material for: Alphacoronaviruses in New World Bats: Prevalence, Persistence, Phylogeny, and Potential for Interaction with Humans
Source: PLoS One. 2011 May 12;6(5):e19156. doi: 10.1371/journal.pone.0019156 (PMC3093381; doi:10.1371/journal.pone.0019156)
Supplement: Figure S1 — Sequence alignment of representative samples of the 1b gene obtained in this study (2007–09 collection) compared with sequences obtained from bats collected in previous study (2006 collection). A. Amplicons obtained from a big brown bat (07-453 EF) and a long legged (07-433 MV) bat in 2007 have 97% sequence similarity with a big brown bat (RM Bt-CoV 65) collected in 2006. B. Amplicon from a long legged bat (07-607 MV) is most similar to amplicons obtained from an occult myotis bat (RM-Bt-CoV 48), but with only 85% similarity. (DOC) [file pone.0019156.s001.doc]

**Supplemental Figure # 1A.**

**Supplemental Figure #1B.**
