# Supplementary material for: Alphacoronaviruses in New World Bats: Prevalence, Persistence, Phylogeny, and Potential for Interaction with Humans
Source: PLoS One. 2011 May 12;6(5):e19156. doi: 10.1371/journal.pone.0019156 (PMC3093381; doi:10.1371/journal.pone.0019156)
Supplement: Table S1 — Primers and RT-PCR programs. A. Consensus primers targeted a highly conserved region of the S2 region of the spike gene and from an exact sequence obtained from one of the big brown bats. PCR was performed under the following conditions: one µL of cDNA was amplified in a 50-µL reaction containing, 0.2 µmol/L deoxynucleoside triphosphates, 1 U of PhusionTaq High-Fidelity DNA Polymerase (Finnzymes, Espoo, Finland), and 2.0 µmol/L primers by the following PCR program: 30 sec at 98°C; 40 cycles for 10 sec at 98°C, 15 sec at 50–52°C (depending on the primer set), and 15 sec at 72°C; and then 10 min at 72°C. B. Primers used for detection of CoV sequence in bat samples. One microliter of cDNA was amplified in a 50-µL reaction containing 1.5 mmol/L MgCl2, 0.2 µmol/L deoxynucleoside triphosphates, 2.5 U of HotStarTaq (QIAGEN), and 2.0 µmol/L primers using the following PCR program: 15 min at 95°C; 45 cycles for 1 min at 95°C, 1 min at 48°C for MY-F and MY-R and 50°C for EF-F and EF-R, and 1 min at 72°C; and 10 min at 72°C. C. To obtain additional sequences for phylogenetic analysis, for two of the CDPHE intestinal samples, RT-PCR was performed using consensus degenerate primers from several areas within the RdRp gene in a SuperScript III one-step RT-PCR system with Platinum Taq High Fidelity kit (Invitrogen, San Diego, CA, USA). Primers and protocols were kindly provided by Suxiang Tong, PhD and Ying Tao, PhD of the Centers for Disease Control and Prevention, Atlanta, Georgia, USA. (DOC) [file pone.0019156.s002.doc]

**Supplemental Table 1a: Spike Primers**

| **Primer Name** | **Fwd/Rev** | **Sequence** |
| --- | --- | --- |
| SpikeF1 | F | 5' TAT GGN TTY TGT GGN AAT GG 3' |
| Spike 597-580 | F | 5' TAA CTC TGC ACC AGA AGG 3' |
| Spike 764-745 | F | 5' CAC ACC TCG TAC CAT GTT TC 3' |
| Spike 41-22 | R | 5' AAA CCA CCT AGT GCC ATA CC 3' |
| Spike 404-386 | R | 5' CGG TCT ACT TGT GCA TCA G 3' |
| Spike 438-421 | R | 5' TGT GCG ACA AAG GCA TTC 3' |
| Spike 998-979 | R | 5' GCT GTT CAA GGT CAG CTA TC 3' |
| JMD-R | R | 5' CCA HAC CCA CCA VGS C 3' |

**Supplemental Table 1b: Screening Primers**

| **Primer name** | **Sequence (5' - 3')** | **Bat Species** | **CoV Gene** | **Tm** | **Amplicon Size** |
| --- | --- | --- | --- | --- | --- |
| EF-F | AAATGAGCTGGCTCAAGTG | *Eptesicus fuscus* | RdRp | 50^o^C | 225 bp |
| EF-R | CGRCGCTGAATACTCTTAAC | *Eptesicus fuscus* | RdRp |  |  |
|  |  |  |  |  |  |
| My-F | GGTTGGGACTATCCTAAGTG | *Myotis* spp. | RdRp | 48^o^C | 179 bp |
| My-R | GAGGTAGAAACCACCATTAG | *Myotis* spp. | RdRp |  |  |
|  |  |  |  |  |  |
| EF-SF1 | TAACTCTGCACCAGAAGG | *Eptesicus fuscus* | spike | 51°C | 418 bp |
| EF-SR1 | GCTGTTCAAGGTCAGCTATC | *Eptesicus fuscus* | spike |  |  |

**Supplemental Table 1c: Replicase Primers**

| **Fragment** | **Primer name** | **Sequence (5’🡪3’)** | **Amplicon Size** |
| --- | --- | --- | --- |
| #1 – 1^st^ round | CoVg1Orf1bF13547, | ATC AGG ACT CTT AIG GTG GNG CNT C | 2 Kb |
|  | CoVg1orf1bR15840, | ATG TTA AAI GCA GAI TTN GCR TAN GC |  |
| #1 – 2^nd^ round | CoVg1orf1bF13800, | GGG GCT CTA GTG CIK CTN GAY TAG | 2 Kb |
|  | CoVg1orf1bR15790, | ACC ACC AGG TTT IAA ATA RAA NCC NCC |  |
| #2 – 1^st^ round | CoVg1orf1bF19340, | CAT GAT ATT GGI AAY CCN AAR GG | 2 Kb |
|  | CoVg1orf1bR21570, | CAG AAA IGC TTC TGA TGA NGA NGT RTT |  |
| #2 – 2^nd^ round | CoVg1orf1bF19487, | TGG AAT TGT AAT GTI GAY ATG TAY CC | 2 Kb |
|  | CoVg1orf1bR21570, | CAG AAA IGC TTC TGA TGA NGA NGT RTT |  |
