# Supplementary material for: Alphacoronaviruses in New World Bats: Prevalence, Persistence, Phylogeny, and Potential for Interaction with Humans
Source: PLoS One. 2011 May 12;6(5):e19156. doi: 10.1371/journal.pone.0019156 (PMC3093381; doi:10.1371/journal.pone.0019156)
Supplement: Text S1 — Influence of different sampling and analysis techniques on CoV RNA detection. (DOC) [file pone.0019156.s003.doc]

**Supplemental Information #1: Influence of Different Sampling and Analysis Techniques on CoV RNA Detection**

From a substantial number of the bats sampled in 2007 we collected multiple types of samples (feces and anal/rectal swabs) in either *RNALater*, M4 VTM, or both. This allowed comparison of various sampling protocols. Using the initial extraction protocol and the conserved primer set for RT-PCR (20), none (0%) of 263 samples collected in *RNALater* and 6 (3.0%) of 201 samples collected in VTM were positive for CoV RNA, including one (0.4%) of 282 anal swabs and 5 (2.3%) of 213 fecal samples. Duplicate samples from 39 bats of *Myotis* spp were placed in *RNALater* and VTM. Five of the VTM samples tested positive for CoV RNA, but none of the samples in *RNALater* were positive for CoV RNA. Similarly, fecal samples and anal swabs collected from 51 bats were compared. Two of the fecal samples were positive for CoV RNA, but none of the anal swabs were positive.

From samples collected in 2007, we randomly chose 30 samples and extracted aliquots of them using either the virus RNA or the universal RNA Tissue EZ1 extraction kits. None of the samples extracted by the virus RNA kit were positive for CoV RNA, but one sample extracted using the universal tissue kit was positive for CoV RNA. RNAs from all 30 samples that had been extracted by the universal tissue kits were then treated using the PCR inhibitor removal kit. Five of the 30 samples were positive for CoV RNA after inhibitor removal, although only one had been positive without treatment to remove PCR inhibitors.

For all 147 of the long-legged bat samples and all 90 of the big brown bat samples, we compared the percent positive for CoV RNA using the conserved consensus coronavirus primer set or primers that were specific to alphacoronavirus RNA sequences found in Rocky Mountain bats of the genus *Myotis* or to big brown bats. Four (1.7%) of the 237 samples were positive using the conserved primer set, whereas 22 (9.3%) were positive using the sequence-specific primer sets.

Currently, there is no standardized method for collecting and analyzing fecal and/or anal swab samples from bats in the wild for virus surveillance studies. Therefore, during the course of this work we experimented with several variations of sample collection to enhance the sensitivity of CoV detection, including differences in sample storage, RNA extraction, removal of PCR inhibitors, and PCR amplification. Detecting CoV RNA in fecal specimens from bats is challenging, likely due to the very small amounts of viral RNA present, the instability of RNA in samples collected in the field, and the possibility of PCR inhibitors in fecal samples. Fecal specimens collected in VTM yielded the highest proportion of samples positive for CoV RNA. Extracting samples using the universal tissue kit, rather than the virus kit, removing PCR inhibitors, and utilizing sequence-specific primers for PCR amplification greatly increased the sensitivity of CoV detection. The use of “lineage-specific” primers to increase surveillance sensitivity was also supported by a recent study in European bats (33). These methodological findings are of importance for several reasons. First, we were unable to re-extract the 2007 samples using the universal RNA tissue kits. Therefore, we may have underestimated the actual prevalence of CoVs in bats sampled at that time. Similarly, because sequence-specific primers greatly increased the sensitivity of our PCR assays, we might not have detected viral RNA of CoVs from bats of other species for which we did not have sequence-specific primer sets. These data should inform future wildlife surveillance projects. They underscore the need for targeted, sensitive, and specific assays for CoV RNA that remove PCR inhibitors that may be present in fecal specimens.
